# Supplementary material for: Strengths and Weaknesses of the Pharmacovigilance Systems in Three Arab Countries: A Mixed-Methods Study Using the WHO Pharmacovigilance Indicators
Source: Int J Environ Res Public Health. 2022 Feb 22;19(5):2518. doi: 10.3390/ijerph19052518 (PMC8909061; doi:10.3390/ijerph19052518)
Supplement: Supplementary file 1 [file ijerph-19-02518-s001.zip › Supplementary file S1.pdf]

PV system strengths and weaknesses in Jordan, Oman, and Kuwait

Interview guide

Pharmacovigilance structure and function

A- Question(s) exploring the structure and function of the pharmacovigilance department or centre

  i- Separate/shared office, number of personnel, facilities available (e.g. database, computers, library), position within hierarchy of organisation, official recognition of department/centre

  ii- Functions being carried out by the department e.g. PV inspections, awareness campaigns, newsletter publication, HCP and/or general public training, additional monitoring and/or additional risk minimisation, involvement in developing treatment guidelines or essential medicines list, collection of data on medicines prescription and consumption

B- Question(s) exploring the structure and function of the country's pharmacovigilance system

  i- Main organisations/individuals involved/operating as part of the system

  ii- Reporting – who reports (physicians, patients, pharmacists, other healthcare professionals, companies), what is reported (type of product e.g. medicines, herbals, supplements, etc., new products only or all products, ADEs or ADRs or both, severity of reactions), what is done with reports e.g. causality assessment, signal detection, statistical analysis

  iii- Existence of peripheral centres – location, interconnectivity and coordination with each other and the main centre; safety communication strategy with stakeholders, advisory committee

iv- Existence of PV advisory committee – representation, decision making process, frequency of meeting

v- PV as part of national curriculum of HCPs

C- Question(s) identifying the legal aspects related to the country's pharmacovigilance system

i- Legal basis for pharmacovigilance - law in the country controlling practice, part of country's medicines policy

ii- Reporting requirements for pharmaceutical companies

iii- Guidelines being followed – existence of local guideline?

Strengths and weaknesses

A- Question(s) exploring participants' opinions on the strengths of the pharmacovigilance department/centre as well as the country's pharmacovigilance system – why and/or how?

B- Question(s) exploring participants' opinions on the weaknesses of the pharmacovigilance department/centre as well as the country's pharmacovigilance system – why and/or how?
