# Supplementary material for: Strengths and Weaknesses of the Pharmacovigilance Systems in Three Arab Countries: A Mixed-Methods Study Using the WHO Pharmacovigilance Indicators
Source: Int J Environ Res Public Health. 2022 Feb 22;19(5):2518. doi: 10.3390/ijerph19052518 (PMC8909061; doi:10.3390/ijerph19052518)
Supplement: Supplementary file 1 [file ijerph-19-02518-s001.zip › Supplementary file S2.pdf]

WHO PV Indicators

Assessment Indicators	Answers	Comments
**Core Indicators**		
**Core Process Indicators**		
1. Total number of ADR reports received in the previous calendar year (also expressed as number of ADRs per 100,000 persons in the population)		
2. Current total number of reports in the national database		
3. Percentage of total annual reports acknowledged and/or issued feedback		
4. Percentage of total reports subjected to causality assessment in the previous calendar year		
5. Percentage of total annual reports satisfactorily completed and submitted to the national pharmacovigilance centre in the previous calendar year Subset indicator: of the reports satisfactorily completed and submitted to the national pharmacovigilance centre, percentage of reports committed to the WHO database		
6. Percentage of reports of therapeutic ineffectiveness received in previous calendar year		
7. Percentage of reports on medication errors reported in the previous year		
8. Percentage of registered pharmaceutical companies having a functional pharmacovigilance system		
9. Number of active surveillance activities initiated, ongoing or completed during the past five calendar years		
**Core Outcome/Impact Indicators**		
1. Number of signals detected in the past 5 years by the pharmacovigilance centre		

2. Number of regulatory actions taken in the preceding year as a consequence of national pharmacovigilance activities includes:<br>a. number of product label changes (variation)<br>b. number of safety warnings on medicines to: (i) health professionals, (ii) general public<br>c. number of withdrawals of medicines<br>d. number of other restrictions on use of medicines		
3. Number of medicine-related hospital admissions per 1000 admissions		
4. Number of medicine-related deaths per 1000 persons served by the hospital per year		
5. Number of medicine-related deaths per 100,000 persons in the population		
6. Average cost (US$) of treatment of medicine-related illness		
7. Average duration (days) of medicine-related extension of hospital stay		
8. Average cost (US$) of medicine-related hospitalization		
**Complementary Indicators**		
**Complimentary Process Indicators**		
1. Percentage of healthcare facilities with a functional pharmacovigilance unit (i.e. submitting ≥ 10 reports to the pharmacovigilance centre) in the previous year		
2. Percentage of total reports sent in the previous year by the different stakeholders includes:<br>a. medical doctors<br>b. dentists<br>c. pharmacists<br>d. nurses or midwives<br>e. the general public<br>f. manufacturers		
3. Total number of reports received per million population per year		

4. Average number of reports per number of health-care providers per year includes:<br>a. medical doctors<br>b. dentists<br>c. pharmacists<br>d. nurses or midwives		
5. Percentage of health-care providers aware of and knowledgeable about ADRs per facility		
6. Percentage of patients leaving a health facility aware of ADRs in general		
7. Number of face-to-face training sessions in pharmacovigilance organized in the previous year for:<br>a. health professionals<br>b. the general public		
8. Number of individuals who received face-to-face training in pharmacovigilance in the previous year:<br>a. healthcare professionals<br>b. the general public		
9. Total number of national reports for a specific product per volume of sales of that product in the country (product specific) from the industry		
10. Number of registered products with a pharmacovigilance plan and/or a risk management strategy among the marketing authorization holders in the country<br>Subset Indicator: Percentage of registered products with a pharmacovigilance plan and/or a risk management strategy from the market authorization holders in the country		
11. Percentage of market authorization holders who submit periodic safety update reports to the regulatory authority as stipulated in the country		

12. Number of products voluntarily withdrawn by market authorization holders because of safety concerns in the previous year<br>Subset Indicator: Number of summaries of product characteristics (SPCs) updated by market authorization holders (MAHs) because of safety concerns in the previous year		
13. Number of reports from each registered pharmaceutical company received by the pharmacovigilance centre in the previous year		
**Complimentary Outcome/Impact Indicators**		
1. Percentage of preventable ADRs reported in the previous year out of the total number of ADRs reported		
2. Number of medicines-related congenital malformations per 100,000 births		
3. Number of medicines found to be possibly associated with congenital malformations in the past 5 years		
4. Percentage of medicines in the pharmaceutical market that are counterfeit/substandard		
5. Number of patients affected by a medication error in hospital per 1000 admissions in the previous year		
6. Average work or schooldays lost due to drug-related problems		
7. Cost savings (US$) attributed to pharmacovigilance activities		
8. Health budget impact (annual and over time) attributed to pharmacovigilance activity		
9. Average number of medicines per prescription		
10. Percentage of prescriptions with medicines exceeding manufacturer’s recommended dose		
11. Percentage of prescription forms prescribing medicines with potential for interaction		

12. Percentage of patients receiving information on the use of their medicines and on potential ADRs associated with those medicines		

Process Indicators: Assess the extent of pharmacovigilance activities (collection, collation, analysis, and evaluation of ADR reports). Assess directly or indirectly the extent to which the system is operating.

Outcome/Impact Indicators: Measure the effects (results and changes) of pharmacovigilance activities. Measure the extent of realization of the pharmacovigilance objectives.
